# Metric structural human connectomes: Localization and multifractality of eigenmodes

**DOI:** 10.1162/netn_a_00439

**Published:** 2025-05-08

**Authors:** Anna Bobyleva, Alexander Gorsky, Sergei Nechaev, Olga Valba, Nikita Pospelov

**Affiliations:** Department of Biophysics, Faculty of Biology of the Moscow State University, Moscow, Russia; Institute for Information Transmission Problems RAS, 127051 Moscow, Russia; Laboratory of Complex Networks, Center for Neurophysics and Neuromorphic Technologies, Moscow, Russia; LPTMS, CNRS – Université Paris Saclay, 91405 Orsay Cedex, France; Department of Applied Mathematics, Higher School of Economics, Moscow, Russia; Institute for Advanced Brain Studies, Moscow State University, Moscow, Russia

**Keywords:** Structural connectome, Network model, Spectral graph theory, Anderson localization, Multifractality, Brain criticality

## Abstract

We explore the fundamental principles underlying the architecture of the human brain’s structural connectome through the lens of spectral analysis of Laplacian and adjacency matrices. Building on the idea that the brain balances efficient information processing with minimizing wiring costs, our goal is to understand how the metric properties of the connectome relate to the presence of an inherent scale. We demonstrate that a simple generative model combining nonlinear preferential attachment with an exponential penalty for spatial distance between nodes can effectively reproduce several key features of the human connectome. These include spectral density, edge length distribution, eigenmode localization, local clustering, and topological properties. Additionally, we examine the finer spectral characteristics of human structural connectomes by evaluating the inverse participation ratios (IPR_*q*_) across various parts of the spectrum. Our analysis shows that the level statistics in the soft cluster region of the Laplacian spectrum (where eigenvalues are small) deviate from a purely Poisson distribution due to interactions between clusters. Furthermore, we identify localized modes with large IPR values in the continuous spectrum. Multiple fractal eigenmodes are found across different parts of the spectrum, and we evaluate their fractal dimensions. We also find a power-law behavior in the return probability—a hallmark of critical behavior—and conclude by discussing how our findings are related to previous conjectures that the brain operates in an extended critical phase that supports multifractality.

## Author Summary

In this work, we explore the fundamental principles underlying the architecture of the human structural connectome. We use a simple generative model that combines nonlinear preferential attachment with biologically plausible geometric constraints. This model successfully reproduces a remarkable number of brain network properties, linking the overall architecture of the brain to its underlying evolutionary principles. Additionally, we investigate the localization properties of connectome eigenmodes and identify multiple indications of their fractality. Drawing on recent advances in Anderson localization in complex systems, we conjecture that the structural connectome operates in an extended critical phase that supports multifractality.

## INTRODUCTION

Understanding the fundamental principles of brain structural organization, which provide efficient signal propagation, is undoubtedly both a challenging and practically important problem in neuroscience. Currently, the dominant paradigm suggests that short-range correlations prevail, and spatiotemporal processes in the brain can be described in terms of wave dynamics on an underlying graph structure. Within this framework, the key element is the graph Laplacian of the structural connectome, which governs the diffusion-like flow of information through the brain. The flow of information along the structural backbone of the connectome can be linked to functional brain dynamics via the so-called “mass model,” which balances activation and inhibition—see Deco, Jirsa, Robinson, Breakspear, and Friston (2008) and Sporns (2013) for a review.

It is widely recognized that the spectral properties of the Laplacian provide crucial insights into brain structure, establishing a link between the spectrum of the structural connectome and cognitive functions (Bullmore & Sporns, 2009; Fornito, Zalesky, & Bullmore, 2016). Recent developments in this field further highlight the significance of this relationship (Abdelnour, Dayan, Devinsky, Thesen, & Raj, 2018; Aqil, Atasoy, Kringelbach, & Hindriks, 2021; Luppi et al., 2023; Robinson, 2021; Tewarie et al., 2020).

Additionally, an increasing body of research emphasizes the importance of the brain cortex’s metric geometry. For example, studies such as Gabay and Robinson (2017) and Robinson et al. (2016) suggest that brain dynamics are governed by a metric Laplacian. Moreover, in models of exponential networks, like those discussed in Roberts, Perry, Roberts, Mitchell, and Breakspear (2017); Robinson (2019); and X.-J. Wang and Kennedy (2016), a typical spatial scale is introduced through the concept of a cutoff, indicating a finite correlation length. In Robinson (2019), the correlation length was related to the damping coefficient, which governs the large-scale asymptotic of the solution to the deformed wave equation. This suggests that understanding the cortex’s geometry, as represented by its metric properties, is essential for comprehending the dynamics of neural activity in the brain.

Recently, it becomes evident that geometric models tend to outperform other methods in analyzing functional magnetic resonance imaging (fMRI) data (Pang et al., 2023) for both task-related and resting-state brain activity. The neuronal field theory model is particularly notable for its simplicity, requiring only one fixed and one adjustable parameter, in contrast to more complex wave propagation models. The cutoff adjustable parameter—dampening coefficient—is fixed by the best fit of the fMRI data. Unlike previous approaches that focused on brain network connectivity (Damoiseaux & Greicius, 2009), it has been found that long-wavelength modes related to brain geometry—specifically those with wavelengths greater than 60 mm—play a dominant role in propagation processes described by the geometric Laplacian. This establishes a typical correlation length in the cerebral cortex.

In this work, we use spectral methods to explore the impact of metric properties on brain organization, employing two complementary approaches. First, we investigate whether a generative model that incorporates intrinsic scale can reproduce the essential spectral features of structural connectomes. Second, by analyzing the transition between localized and delocalized excitations propagating through a structural connectome, we examine the presence of localized modes in the spectrum, which imply a spatial scale that defines the localization length. When analyzing structural connectomes, it is unlikely that all eigenmodes are completely delocalized, as this would imply that the system is ergodic, making it difficult to observe persistent activity. Conversely, complete localization of eigenmodes would eliminate correlations between brain regions. Thus, the most probable scenario is the coexistence of both localized and delocalized modes. An initial discussion of this topic can be found in Pospelov et al. (2019), though only a limited number of connectomes were analyzed in that study. Therefore, further statistical verification and a more comprehensive analysis are needed to substantiate these claims.

Since spectral analysis is the central part of our study, it is useful to recall its most relevant aspects. In what follows, we will use both the spectra of the Laplacian and the adjacency matrix of the graph.

▪  The spectrum of the graph Laplacian is nonnegative and typically consists of two parts. The first part involves small eigenvalues, which we will refer to as “soft” or “cluster” modes. Their origin is as follows: If a graph has *N* disconnected components, the multiplicity of the zero mode equals *N*. However, if these components are weakly connected, *N* isolated soft modes emerge (Nadakuditi & Newman, 2012). It has been rigorously proven that the number of isolated soft modes in the Laplacian spectrum corresponds to the number of weakly connecting clusters. The second part of the spectrum consists of a continuum of modes with higher eigenvalues, which we will refer to as “bulk” modes. In many cases, the spectral density in the bulk region exhibits a sharp peak at a specific eigenvalue of the Laplacian. This peak corresponds to a sharp peak at *λ* = 0 in the spectral density of the adjacency matrix. Analytically, it has been shown that it signifies strong network heterogeneity and is absent in sufficiently homogeneous graphs (Silva & Metz, 2022).
▪  To investigate the localization properties of propagating modes on the graph, one can analyze specific features of the eigenvalues and/or eigenfunctions (eigenmodes). One of the widely used indicators for this is the level spacing distribution *P*(*s*). If *P*(*s*) follows a Poisson distribution along the whole spectrum, the eigenmodes do not interact, and the system is in a localized regime. On the other hand, if *P*(*s*) exhibits Wigner-Dyson surmise statistics, the system is delocalized and eigenvalue repulsion occurs. If *P*(*s*) demonstrates Poisson behavior in one part of the spectrum and Wigner-Dyson behavior in the rest, the system displays Anderson criticality (Shklovskii, Shapiro, Sears, Lambrianides, & Shore, 1993).
▪  Inverse participation ratio (IPR*_q_*) is a widely used measure to analyze the localization phenomenon in terms of eigenvector properties. An eigenmode with a fractal dimension *D*_*q*_ = 0 indicates a localized mode, while *D*_*q*_ = 1 corresponds to an extended mode. Modes with 0 < *D*_*q*_ < 1 display fractal behavior, and nonlinearity in the variation of *D*_*q*_ with *q* signals the presence of multifractality. For a detailed review, see Evers and Mirlin (2008).

In Pospelov et al. (2019), it was found that within the “soft” part of the Laplacian spectrum corresponding to small eigenvalues associated with clusters (Nadakuditi & Newman, 2012), the level spacing distribution *P*(*s*) follows a deformed Poisson law. However, in the continuous part of the spectrum, *P*(*s*) transitions to a mixed Poisson-Wigner distribution. From the perspective of Anderson localization in systems with diagonal disorder, this behavior suggests that the system operates near a critical regime (Shklovskii et al., 1993). It was suggested that a sharp mobility edge separates localized from delocalized modes. Although the structural connectome lacks on-site diagonal disorder, as found in conventional Anderson models, its intrinsic structural disorder plays a similar role. To confirm the presence of criticality and a mobility edge, a more detailed analysis of individual Laplacian and adjacency eigenmodes is necessary. One direct approach for addressing this is to examine the IPR of all eigenmodes. Elevated IPR values indicate localization, and our aim is to clarify the localization properties of these modes within the structural connectome.

The presence of localized modes in the structural connectome spectrum was noted in Moretti and Muñoz (2013), where it was proposed that the connectome operates not at criticality, but rather in a Griffiths phase. This is more plausible from a functional perspective, as it does not require the fine-tuning of parameters that a purely critical state would demand. In a Griffiths phase, signatures of criticality, such as power-law behavior, appear not just at a specific value of a control parameter, but over a range of values. In the conventional Anderson model, diagonal disorder acts as the control parameter. However, in this case, only structural disorder was considered, and the existence of a Griffiths phase was confirmed in Muñoz, Juhász, Castellano, and Ódor (2010) and Ódor, Dickman, and Ódor (2015). It was later proposed (Buendía, Villegas, Burioni, & Muñoz, 2022) that a second form of criticality expected in the brain—the synchronization phase transition—could also expand into a Griffiths phase. Various generic aspects of brain criticality are discussed in reviews—see, for example, Beggs (2022); Cocchi, Gollo, Zalesky, and Breakspear (2017); Grosu et al. (2023); Hesse and Gross (2014); and O’Byrne and Jerbi (2022).

In this study, we explore another spectral characteristic of the structural connectome that was not addressed in Moretti and Muñoz (2013) and Pospelov et al. (2019)—multifractality of eigenmodes (for a review, see Evers & Mirlin, 2008). Multifractality is a property of the intermediate phase between the ergodic and deterministic phases. It can also be considered an extension of Anderson criticality (Janssen, 1994). While multifractality was initially thought to be a feature unique to the critical point, more recent research has shown that it can also manifest in the extended phase. This phase is typically referred to as the non-ergodic extended (NEE) phase or the extended multifractal phase. The NEE phase was first identified in the generalized Rosenzweig-Porter model (Kravtsov, Khaymovich, Cuevas, & Amini, 2015) and has been observed in a clear-cut manner in several models with matrix Hamiltonians (Biroli & Tarzia, 2020; Bogomolny & Sieber, 2018; Facoetti, Vivo, & Biroli, 2016; Kravtsov et al., 2015; Monthus, 2017; Monthus & Garel, 2011; Motamarri, Gorsky, & Khaymovich, 2022; Tarzia, 2022).

We are interested in the existence of multifractal modes within the structural connectome, which is a complex network, and there are indeed relevant examples. Multifractality has been observed in the Anderson model with a diagonal disorder on a Cayley tree (Biroli & Tarzia, 2020; Monthus & Garel, 2011; Sonner, Tikhonov, & Mirlin, 2017). It has been shown that the fractal dimension of these modes varies depending on their location on the tree (Sonner et al., 2017). More recently, NEE phase with multifractal eigenmodes was clearly identified in an Erdős-Renyi graph ensemble in the sparse regime above the percolation threshold, even without diagonal disorder (Cugliandolo, Schehr, Tarzia, & Venturelli, 2024). It was suggested that the mechanism behind this multifractality may involve weakly interacting clusters.

In this paper, we focus on the following issues:

*Generative model incorporating spatial intrinsic scale*. We explore the principles behind the construction of a graph that is similar to a connectome and could yield experimentally verifiable spectral density. For structural connectomes, we consider the generative model that employs nonlinear preferential attachment and an exponential parameter-dependent cutoff in edge lengths. This approach reproduces the geometric length distribution of fiber lengths found in the connectome as well as the spectral features and clustering properties. Similar generative models combining metric aspects and preferential attachment in a general network framework have been introduced in Zuev, Boguná, Bianconi, and Krioukov (2015). In brain networks context, this type of generative models has been considered in Betzel et al. (2016) and Vértes et al. (2012) and different aspects of such models have been discussed in Akarca et al. (2022, 2023); Akarca, Vértes, Bullmore, CALM team, and Astle (2021); Goulas, Betzel, and Hilgetag (2019); and Oldham et al. (2022). The recent reviews on the generative models of connectomes can be found in Astle, Johnson, and Akarca (2023) and Betzel and Bassett (2017).
*IPR and localization of eigenfunctions*. We investigate the IPR that indicates localization properties of Laplacian or adjacency matrix eigenmodes. Large IPR values for some eigenmodes imply that these modes are localized in certain graph regions, while small values indicate delocalized states. Generically, there are two types of coexistence between localized and delocalized states: the existence of a mobility edge separating these states, and the presence of “scars” (Chandran, Iadecola, Khemani, & Moessner, 2023) corresponding to isolated localized states within the delocalized part of the spectrum. Our findings are as follows: There are states with high IPR values in the low-energy region of the spectrum that are localized at clusters, in agreement with Pospelov et al. (2019). In addition, there are localized states at large eigenvalues of Laplacian with large IPR that form two distinct small bands around the eigenvalues *λ* = 0 and *λ* = −1 of the adjacency matrix. These bands correspond to topologically equivalent nodes (TENs), similar to patterns observed in Kochergin, Khaymovich, Valba, and Gorsky (2023). Some of *λ* = 0 modes are trivial TENs, which are the endpoints of the graph leaves. Therefore, despite the presence of localized states in the bulk, there is no sharp mobility edge in the bulk as suggested in Pospelov et al. (2019). However, one could speak of an effective mobility edge between the bulk and the band of cluster-isolated modes.
*Multifractal eigenmodes*. Multifractality is another potential localization-related statistical pattern that can be identified through the analysis of the *q*-dependent fractal dimensions *D*_*q*_. These dimensions are defined by the variation of the IPR*_q_* over different scales (see Equation 9). We analyzed *D*_*q*_ across various parts of the spectrum and unexpectedly found multiple eigenmodes with fractal dimension in the range *D*_*q*_ = 0.7 − 0.8, some of them with weak multifractality. These features of multifractality include the weak nonlinearity of the fractal dimension and the power-law scaling of random walk return probabilities.
*Correlation of clusters*. Modes of Laplacian with small eigenvalues exhibit a semi-Poisson distribution, which is consistent with the general arguments presented in Avetisov, Gorsky, Nechaev, and Valba (2020). The deviation of the level spacing distribution *P(s)* from a pure Poisson distribution indicates interactions among cluster modes, similar to those extensively studied in Kochergin et al. (2023). It is noteworthy that recent spectral analysis of the *C. elegans* structural connectome demonstrates the precise identification of clusters as the soft modes of Laplacian even in sparse scenarios (Onuchin, Chernizova, Lebedev, & Polovnikov, 2023). To substantiate the presence of cluster interactions, we examine the correlations between the lowest eigenvalues (*λ*_2_, *λ*_3_, *λ*_4_). Given that the identification of *λ*_2_ is straightforward—it quantifies the number of links between hemispheres M. B. Wang, Owen, Mukherjee, and Raj (2017)—we investigate the behavior of (*λ*_3_, *λ*_4_) as a function of *λ*_2_.

The paper is structured as follows. In Description of the Nonlinear Geometric Model, we introduce the generative model for the structural connectome. In NGPA Model Versus Real Connectomes, we compare various spectral characteristics of the model with experimental data. Statistics of Eigenmodes analyzes the eigenmode statistics and proposes a conjecture that the structural connectome operates in an extended non-ergodic multifractal phase. The findings are summarized in the Discussion section.

## DESCRIPTION OF THE NONLINEAR GEOMETRIC MODEL

Nowadays, the Watts-Strogatz model Watts and Strogatz (1998) is considered as a good “baseline” network that adequately describes many experimentally observed features of the brain’s structural network. This model has a “small world” network structure, which combines short optimal paths with a high clustering coefficient. This interplay between the two properties is crucial for efficient brain function, providing significant advantages in signal processing. This structural organization is essential for healthy brain function: Indeed, deviations from these “small world” features have been observed in groups of patients with Alzheimer’s disease, autism, and schizophrenia, as reported in Hilgetag and Goulas (2016).

Since the pioneering work (Friston, 2008), more and more authors have proposed the “hierarchical” organization of the functional brain connectomes (see, e.g., Akiki & Abdallah, 2019; Meunier, Lambiotte, Fornito, Ersche, & Bullmore, 2009). However, whether structural connectomes also have hierarchies consistent with functional ones remains an open question (Smith et al., 2019; R. Wang et al., 2019).

Recent studies (Pang et al., 2023) provided convincing evidence that the agreement between experimental data and mathematical models improves significantly when the metric structure of structural connectomes is taken into account. An interesting example are metric-aware generative models of axonal outgrowth that produce artificial networks with topological properties similar to those of real connectomes (Liu et al., 2024; Song, Kennedy, & Wang, 2014). Changes in brain network structure, in general, and changes in connection lengths, in particular, are linked to neurodegenerative diseases (Alexander-Bloch et al., 2013; Gollo et al., 2018).

One powerful approach to study network properties is to consider a family of “null models”—artificial network counterparts with some information washed out from them (Váša & Mišić, 2022). However, here, we wish to reconstruct various network properties from structural connectomes using the “first principles.” Therefore, we set growth rules for artificial networks instead of applying some sort of randomization to original ones.

Here, we discuss a simple model that has the desired properties: (a) small-world behavior, (b) a high clustering coefficient, and (c) a spatial cutoff due to being embedded in a real three-dimensional space.

Specifically, we consider a model that combines the abstract generalized preferential attachment algorithm with an exponential penalty for long edges in a graph constructed from 3-dimensional coordinates of a human connectome. As it has been shown in Krioukov, Papadopoulos, Kitsak, Vahdat, and Boguñá (2010), the preferential attachment models with a scale-free node degree distribution (and hence with a high clustering coefficient) allow for a natural embedding into the hyperbolic Poincaré disc of finite radius. Due to their hyperbolic nature, scale-free networks are closer to trees than exponential Erdős-Rényi graphs. However, the scale-free networks created in this way are purely topological and do not contain any information about the spatial proximity of nodes in structural connectomes that exist in 3D space. The information about the metric structure of our model network is derived from the actual coordinates of nodes in human brain connectomes and is then applied to an artificial network created using the preferential attachment algorithm. This model is referred to as the nonlinear geometric preferential attachment (NGPA) model. NGPA model creates nonweighted graphs and deals with symmetric adjacency matrices. It belongs to the family of models suggested in Betzel et al. (2016) and Zuev et al. (2015).

### NGPA Algorithm

Our network construction algorithm consists of the following steps:

1. Select and preprocess (as described in the section “Human connectome data” in the Methods section) a specific human structural connectome with *N* nodes and *E* edges from the dataset of structural human connectomes (Kerepesi, Szalkai, Varga, & Grolmusz, 2017);
2. The network construction process starts with an empty network with *N* nodes. We preserve the 3D coordinates (*x*, *y*, *z*) of all connectome nodes and remove all existing edges. Note that the set of 3D node coordinates is the whole information inherited from the sample of the original structural human connectome that enables us to create its metric-dependent artificial counterpart. To test the impact of precise node positions on the results, we have run experiments for “pseudoconnectomes” with randomly positioned nodes taken from the uniform distribution in each hemisphere. Spectra and lengths’ distribution computed for “pseudoconnectomes” turned to be similar to those for real connectomes, suggesting that precise spatial positions of nodes do not affect significantly the spectral and geometric characteristics of the model;
3. Create a new (artificial) set of edges (instead of the removed one) based on a preserved set of all known (*x*, *y*, *z*) coordinates of nodes. We proceed with the incremental model construction by selecting the network nodes one by one and growing new edges for them. This process is described by the generalized preferential attachment algorithm, which has two components: topological and metric and is described below.
  (a)  *Topological component of preferential attachment*. Proceeding inductively, suppose that *G* is a new partially constructed artificial network with some nodes connected by newly formed edges. Let node *j* in the network be connected to *d*_*j*_ other nodes by new edges. Now, a new node *i* is selected and connected to node *j* with a probability that depends on the degree of node *j*, following the preferential attachment algorithm. Specifically, the probability $P_{ij}^{\left(PA\right)}$ of forming a link *ij* is given by:
    $$P_{ij}^{\left(PA\right)}=\frac{{\left(d_{j}+1\right)}^{\alpha }}{\sum_{k=1}{\left(d_{k}+1\right)}^{\alpha }}$$ (1)
    where *α* ≥ 0 is the parameter of the model (*α* = 1 corresponds to the standard “rich gets richer” linear preferential attachment model; Barabási, 2013). We have added 1 to every *d*_*j*_ in order to be able to define a nonzero connection probability for isolated vertices (i.e., when *d*_*j*_ = 0). For *α* ≠ 1, the preferential attachment model is nonlinear.
  (b)  *Metric component of preferential attachment*. To construct a graph that inherits the structure of a real connectome embedded in a 3D space, we penalize long links formation by introducing an exponential cutoff. Namely, if the Euclidean distance between the node *j* and a newly added node *i* is $r_{ij}=\sqrt{{\left(x_{i}-x_{j}\right)}^{2}+{\left(y_{i}-y_{j}\right)}^{2}+{\left(z_{i}-z_{j}\right)}^{2}}$, then the probability of a link *ij* formation is multiplied by the factor *e*^−*r*_*ij*_/*r*_0_^, where *r*_0_ (*r*_0_ > 0) is the cutoff parameter. We select *r*_0_ as *r*_0_ = *l*_0_/*β*, where *l*_0_ = 〈*r*_*ij*_〉 = ∑_*i, j*_*r*_*ij*_/*E* ≈ 15 mm (recall that *E* is the number of links in the network) is the average link length in the selected human connectome.
    By combining the topological and metric components defined above, we construct the probability *p*_*ij*_ of the formation of a link *ij* of a network embedded in the three-dimensional space according to the following rule:
    $$p_{ij}=\frac{Q_{ij}}{\sum_{j}^{N}Q_{ij}},\;Q_{ij}=P_{ij}^{\left(PA\right)}e^{-\beta r_{ij}/l_{0}}=\frac{{\left(d_{j}+1\right)}^{\alpha }}{\sum_{k=1}{\left(d_{k}+1\right)}^{\alpha }}e^{-\beta r_{ij}/l_{0}}$$ (2)
    The recursive procedure runs as follows. In the first step, only solitary nodes positioned at specific locations in 3D space are present. In the next step, we choose a random node *i* and connect it to *m*_*i*_ ∈ ℕ randomly selected vertices, taking into account the degrees of other nodes and the penalty for excessively long edges (see Equation 2). Here, *m*_*i*_ is a random variable uniformly distributed within the interval $\left[1,\frac{2E}{N}\right]$ (recall that *E* and *N* are the total number of edges and nodes in the final graph, respectively, and therefore, $\frac{2E}{N}$ is the average vertex degree). We then repeat this process for each node in the graph, with nodes selected in random order. Throughout the algorithm, no target node is selected more than once (double connections are excluded). By randomly selecting the number of new connections, *m*, within the range $\left[1,\frac{2E}{N}\right]$ on each step, we ensure that the final network has approximately the same density of edges as the original human connectome.
4. Described network construction algorithm is applied separately to sets of nodes from the left and right hemispheres, and then *E*_*interhem*_ interhemispheric connections are added to connect the two hemispheres of the model. These connections are created by randomly selecting a pair of nodes, one from each hemisphere. Although the number of interhemispheric connections *E*_*interhem*_ is small compared with the total number of connections *E*, they are excluded from the analysis of edge lengths (only connections within each hemisphere are considered).

### Connection With Other Models

For brevity, we will refer to the NGPA model as not only the result of the random network generation process described above but also as the entire pseudoconnectome *M*(*α*, *β*), which consists of two model “hemispheres” (NGPA graphs) and their interhemispheric connections.

Note that standard network models can be derived through the two-parameter NGPA process with specific parameter values. For example, selecting *α* = 0 and *β* = 0 (*M*(0, 0)) results in an Erdős-Rényi-like random graph (Erdős & Renyi, 1960). Selecting *α* = 0 and *β* > 0 imposes soft geometric constraints on network connectivity as in the random geometric graph model (but without a hard boundary). The limiting model *M*(0, ∞) is approximately a *k*-nearest neighbors graph (since only the closest nodes are connected). On the other hand, NGPA models with preferential attachment and no geometric constraints (*α* > 0 and *β* = 0) correspond to Barabási-Albert-like graphs, as described in Onody and de Castro (2004). These graphs have different regimes: *α* < 1 corresponds to the subcritical regime, *α* = 1 to the standard scale-free network, and *α* > 2 to the “winner-takes-all” regime (Barabási, 2013).

The idea of combining wiring cost optimization and some kind of attractive force between network nodes is not new and has a fruitful history in connectome modeling. In Goulas et al. (2019), a growth model was proposed that encourages homophilic connections and punishes long-distance connectivity.

The closest to NGPA model are the generative models proposed in Akarca et al. (2021) and Betzel et al. (2016). These models calculate the probability of connections directly by multiplying a “geometry penalty term” and a “node homophily term.” The definition of these terms may vary significantly, with the authors of Betzel and Bassett (2017) studying 13 different implementations. In Akarca et al. (2022), the authors created a generative model for neural networks using the same probabilistic structure for growing connections. Vértes et al. (2012) also used this approach for functional brain networks.

Note that NGPA uses exponential penalties for long-range connections instead of power laws, which is consistent with arguments presented by Oldham et al. (2022), and has been confirmed by biological studies on connectome spatial embedding (Ercsey-Ravasz et al., 2013; Horvát et al., 2016). Despite this and several other minor implementation details, the NGPA model differs from the aforementioned models in two main ways:

1. The NGPA model is constructed separately for each hemisphere, dealing only with intrahemispheric connections. This is because we aim to maintain the two-hemisphere brain structure and closely examine the interaction between clusters from different parts (see the “Correlation of Cluster Modes” section). Additionally, interhemispherical connections are more conserved and genetically determined, although this is an area of ongoing research (Wainberg et al., 2024). These long-distance connections play a significant role in brain function; for example, it has been shown that large, myelinated fibers crossing the corpus callosum are necessary to form bilateral functional connections (Shen et al., 2015). Therefore, we decided not to include them in the random growth process.
2. Random generative models mentioned above construct specific energy metrics by taking into account a large number of network properties simultaneously, such as clustering, efficiency, modularity, and degree distribution. Optimal parameter selection procedures for such models include efficient sampling of simulated networks (Liu et al., 2023), parameter space partitioning with Voronoi tessellation (Betzel et al., 2016) and simulated annealing (Vértes et al., 2012). This contrasts with our approach: In the first stage of fitting the NGPA model parameters, we only care about the edge length distribution, while in the second stage, we focus on spectral correspondence. Moreover, during the optimization process, the search space is significantly reduced, as we only look for networks that meet strict geometric constraints during the second phase. Details of our parameter selection process can be found in the “Selecting Optimal Parameters” section.

However, the NGPA model is able to reproduce local clustering, topological overlap, the number of structural clusters, and many other characteristics that we report below. We consider the simplicity of the fitting procedure to be an important advantage of our algorithm, as our ultimate goal is to identify which network characteristics are truly necessary to replicate a plethora of network phenomena observed in real connectomes.

## NGPA MODEL VERSUS REAL CONNECTOMES

### Spectral Density and Topological Overlap

We apply the NGPA model, as formulated above, to the real-world structural connectome data. To begin, we focused on the simplest spectral characteristic—the spectral density, which can be defined for the graph Laplacian *L* as follows:

$$\rho \left(\lambda \right)=\frac{1}{N}\sum_{i}\delta \left(\lambda -\lambda_{i}\right)$$ (3)

where *λ_i_* is the *i*-th eigenvalue of the Laplacian matrix *L*. Recall that the graph Laplacian is related to the adjacency matrix of the graph *A* via the equation *L* = *D* − *A*, where *D* is a diagonal matrix with the degrees of the nodes on the diagonal. The normalized Laplacian, *L*^norm^ = *D*^−1/2^*LD*^−1/2^, is often used to control the influence of hubs in collective dynamics, especially in heterogeneous networks such as connectomes. The normalized Laplacian matrix has several advantages. Its spectrum always lies in the interval *λ* ∈ [0, 2], so it can be used to compare networks with different sizes. Additionally, all eigenvalues of both *L* and *L*^norm^ are nonnegative, with *λ*_1_ = 0 and all other eigenvalues *λ_i_* > 0. This property holds for connected graphs and is ensured by our preprocessing and network construction procedures (see Methods section).

To test the proposed model, we compared the spectral features of the connectomes with simpler models that were obtained by removing some components from the original NGPA model *M*(*α_opt_*, *β_opt_*). These models included: a random network *M*(0, 0), a model without geometric penalty *M*(*α_opt_*, 0), and a model with no preferential attachment *M*(0, *β_opt_*(0)).

**Figure 1.**
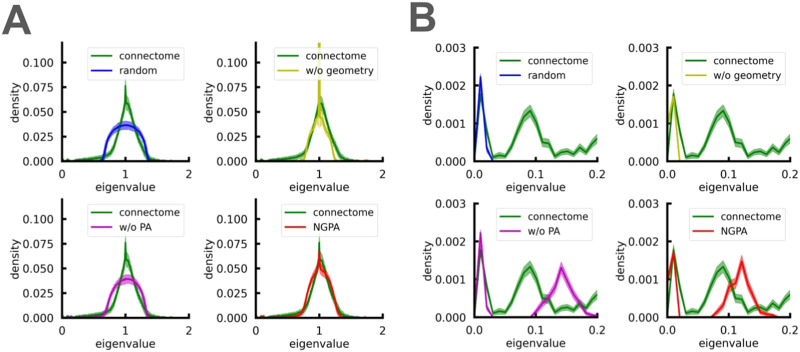
Spectral density of normalized Laplacian matrices for real connectomes and models. (A) full spectrum, (B) insets for low-energy regions of the spectra. Models shown: “random” = *M*(0, 0), “w/o geometry” = *M*(*α_opt_*, 0), “w/o PA” = *M*(0, *β_opt_*(0)), “NGPA” = *M*(*α_opt_*, *β_opt_*). Spectra are averaged over 100 connectomes and corresponding models. Shaded regions indicate standard errors. EMDs between distributions are reported in Table 1.

Spectral densities of the models and their comparison with spectral density of connectomes are shown in Figure 1A. Note that both a random model and a geometry-aware model have a semicircle-shaped spectral density, characteristic of simple random graphs (Erdős, Knowles, Yau, & Yin, 2013; more precisely, the limiting spectral density is the convolution of a standard Gaussian distribution with Wigner’s semi-circular law [Ding & Jiang, 2010], since we are interested in the spectrum of *L*^norm^). The only significant difference between these two models is the presence of a low-energy eigenvalue region in the geometry-aware model, which corresponds to the natural formation of connected clusters under geometric constraints (Figure 1B). However, this zone is significantly shifted to the right compared with the real spectra, indicating weaker cluster structure.

**Figure 2.**
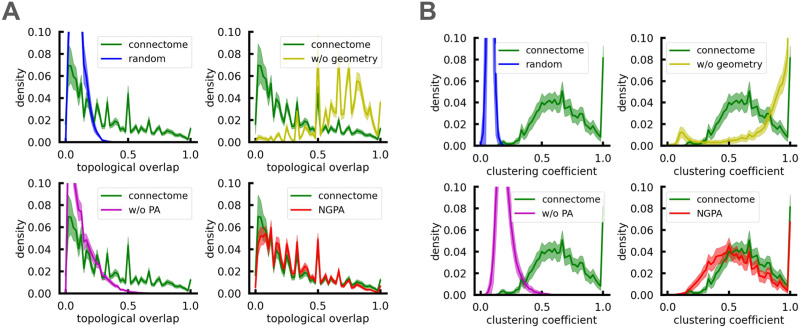
(A) Distributions of topological overlap matrix elements in the real connectomes and the models. (B) The same for local clustering coefficients distributions. Curves are averaged over 100 connectomes and corresponding models; shaded regions indicate standard errors. Models shown: “random” = *M*(0, 0), “w/o geometry” = *M*(*α_opt_*, 0), “w/o PA” = *M*(0, *β_opt_*(0)), “NGPA” = *M*(*α_opt_*, *β_opt_*). EMDs between distributions are reported in Table 1.

As for the model with preferential attachment but without geometric constraints (*M*(*α_opt_*, 0)), its spectral density has a more triangular shape due to the scale-free structure (Farkas, Derenyi, Barabási, & Vicsek, 2011). Together with the NGPA model, they provide a better approximation to the spectral density of the bulk than homogeneous models. This bulk shape is also typical for structural connectomes of other organisms, including cats and monkeys (de Lange, de Reus, & van den Heuvel, 2014). However, one important difference is found in the low-energy region: While *M*(*α_opt_*, 0) has no such eigenvalues (except for one separated eigenvalue *λ*_2_, which is always present in two-hemispheric graphs), the NGPA model shows a clear peak in the low eigenvalue density, which is close to that in real connectomes. Note that the triangular form of the spectral density for *M*(*α_opt_*, 0) model is not a surprise since it is characteristic of any model with the scale-free degree distribution. This model does not produce nontrivial clusters (and thus low-magnitude eigenvalues) due to the absence of geometric constraints. However, nonlinear preferential attachment results in high local clustering coefficients (Figure 2B).

Typically, the Laplacian spectrum consists of several isolated soft (low-energy) modes corresponding to clusters, as described in Nadakuditi and Newman (2012). These are accompanied by continuous bulk modes. A comparison between the numerical data and the NGPA model in Figure 1 shows that the spectral density of the normalized Laplacian *L*^norm^ in NGPA closely resembles the bulk spectral density calculated from experimental data. The number of isolated eigenvalues (*λ* < 0.15) corresponding to clusters is also correctly reproduced (4.3 ± 0.6 for the connectomes, 3.9 ± 0.2 for corresponding NGPA models), but their positions show slight shifts compared with those in the real connectome (see Figure 1B).

Note that both the *M*(*α_opt_*, *β_opt_*) (NGPA) model and the *M*(*α_opt_*, 0) (geometry-free) model have a peak in the density of eigenvalues around *λ* = 1, as shown in Figure 1A. This peak in the Laplacian spectrum corresponds to a peak in the adjacency matrix spectrum at *λ* = 0. These peaks in Laplacian and adjacency matrix spectrum are indicative of a high level of heterogeneity in the graph (Silva & Metz, 2022). When a parameter is introduced that effectively measures heterogeneity, there is a transition at some point between spectra with and without peaks. The intuitive explanation for this is as follows: More heterogeneous networks have highly skewed degree distributions, which results in the presence of nodes with similar connectivity, or TENs. These nodes produce eigenvalues close to = 0 (due to degeneracy of their connectivity). We discuss this issue further in the Statistics of eigenmodes section in terms of adjacency matrix eigenmodes localization. Therefore, we can conclude that the structural connectome falls into the category of heterogeneous networks.

Among the random models considered, only the NGPA model accurately reproduces this peak, while the geometry-free model significantly overestimates it (its spectral density in Figure 1 is truncated from above for better visibility). This is because the geometry-free approach can lead to the formation of superhubs forcefully attracting connections from weaker nodes, which leads to highly heterogeneous structure.

It is instructive to compare the NGPA model with the null model proposed in Pospelov et al. (2019), which also reproduces the spectral density reasonably well. Both models highlight the significance of the local structure of the connectome. In the NGPA model, local properties are incorporated through the exponential cutoff of edges that significantly exceed *r*_0_ in length, imposing a soft spatial constraint on the preferential attachment process. Conversely, the null model proposed in Pospelov et al. (2019) addresses local properties by introducing additional constraints associated with the local clustering that govern network evolution. The number of such additional constraints is proportional to network size *N*. In both cases, numerous local constraints suppress network growth. It is worth noting, however, that in the NGPA model, this behavior is achieved by controlling a single tuning parameter *r*_0_, which defines the geometric scale. Combined with the nonlinear preferential attachment mechanism, it is sufficient to reproduce quantitatively the distribution of local clustering coefficients of nodes (Figure 2B). This means that high transitivity of brain’s structural networks and even local clustering structure, which was important in Pospelov et al. (2019), can be obtained as a by-product of the combination of the two structural principles outlined above.

The NGPA model also reproduces well the topological overlap matrix, *O*_*ij*_ of a real connectome. Following Ravasz, Somera, Mongru, Oltvai, and Barabási (2002), the matrix *O*_*ij*_ is defined as follows:

$$O_{ij}=\frac{C\left(i,j\right)+A_{ij}}{\min\left(k_{i},k_{j}\right)+1-A_{ij}},$$ (4)

where *C*(*i*, *j*) is the number of common neighbors of node *i* and *j, A*_*ij*_ is the corresponding adjacency matrix element (*A*_*ij*_ = 1 if there is a direct link between *i* and *j* and 0 otherwise), and *k*_*i*_ is the degree of node *i*. A topological overlap of 1 between nodes *i* and *j* implies that they are connected to the same vertices, whereas a 0 value indicates that *i* and *j* do not share links to common nodes among the nearest neighbors. The corresponding distributions of *O*_*ij*_ elements is shown in Figure 2A. Note that the peaks of this distribution correspond to a high number of linked node pairs with disjoint common neighbor sets (or, alternatively, node pairs having a single common neighbor): $O_{ij}=\frac{1}{2},\frac{1}{3},\frac{1}{5}$, and so forth. The NGPA model correctly reproduces these peaks, at the same time slightly overestimating their amplitude. We attribute this to the preferential attachment mechanism acting on the scales smaller than *r*_0_ and thus not being suppressed by the geometric penalty, which stimulates formation of “many-to-one” connectivity patterns. On big spatial scales, the geometric penalty dominates, and the effects of preferential attachment (PA) are ruled out, in contrast with the PA-based model. At the same time, the geometry-based model does not incorporate preferential attachment effects, which results in systematic underestimation of the topological overlap.

### Hyperbolic Embedding

Hyperbolic random graphs are a promising category of geometric graphs, whose nodes are somehow embedded in the hyperbolic metric space (Gugelmann, Panagiotou, & Peter, 2012). It has been shown that they share many characteristics with complex real-world networks, including a power-law degree distribution, small diameter and average distance, and a high clustering coefficient (Krioukov et al., 2010). Important open questions include determining the embedding of a real network in a hyperbolic space and identifying the signature of a hyperbolic manifold from network data.

One may question why the hyperbolicity of the brain gains the interest of researchers and what insights can be gained from measuring hyperbolicity. It is worth noting that hyperbolic manifolds cannot be embedded in any Euclidean space with a fixed dimension. However, they can be embedded in a space where volume grows exponentially with the radius. These spaces are called “hyperbolic.” The more “hyperbolic” the manifold, the greater the space it explores, but the brain network is constrained by its confinement in the finite three-dimensional cranium.

The conflict between the exponential growth of the network and the limited volume it occupies inevitably leads to the formation of “crumples,” resulting in the emergence of “shortcuts” in the embedded manifold. These shortcuts may create additional connections that reduce the time required for signals to travel from one brain area to another. In other words, one can hypothesize that a more hyperbolic brain operates faster.

The hyperbolicity of a graph was defined by Gromov (1987; we are only discussing here the so-called 4-points condition). Let *v*_1_, *v*_2_, *v*_3_, and *v*_4_ be vertices of a graph and let *S*_1_, *S*_2_, and *S*_3_ be defined as follows:

$$\begin{array}{l} S_{1}=d\left(v_{1},v_{2}\right)+d\left(v_{3},v_{4}\right)\\ S_{2}=d\left(v_{1},v_{3}\right)+d\left(v_{2},v_{4}\right)\\ S_{3}=d\left(v_{1},v_{4}\right)+d\left(v_{2},v_{3}\right) \end{array}$$ (5)

where *d*(*v*_*i*_, *v*_*j*_) is the shortest path length between *v*_*i*_ and *v*_*j*_. Take *M*_1_ and *M*_2_—the two largest values among *S*_1_, *S*_2_, and *S*_3_. We define the hyperbolicity for node set (*v*_1_, *v*_2_, *v*_3_, *v*_4_) as

$$\delta \left(v_{1},v_{2},v_{3},v_{4}\right)=M_{1}-M_{2},$$ (6)

The hyperbolicity *δ*(*G*) of the entire graph is the average overall possible 4-point hyperbolicities:

$$\delta \left(G\right)={\langle \delta \left(v_{1},v_{2},v_{3},v_{4}\right)\rangle }_{v_{1},v_{2},v_{3},v_{4}\in V\left(G\right)}$$ (7)

**Figure 3.**
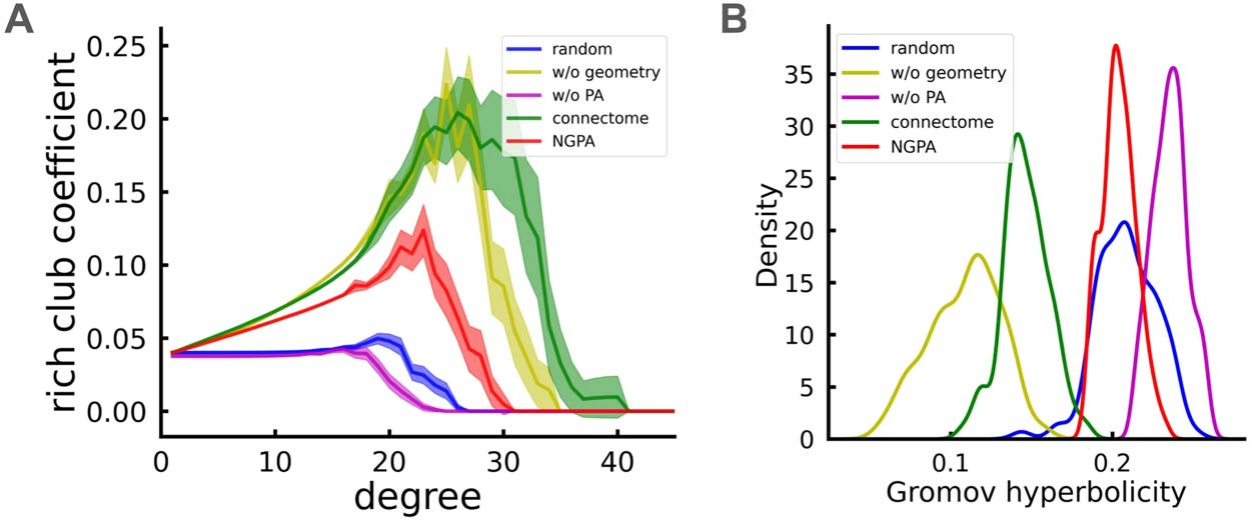
(A) Rich-club coefficients for real connectomes and model networks. The curves are averaged over 100 connectomes and corresponding models. Shaded regions indicate standard errors. (B) Gromov hyperbolicity distributions from the same networks. Note that each network has a single hyperbolicity value. Models shown: “random” = *M*(0, 0), “w/o geometry” = *M*(*α_opt_*, 0), “w/o PA” = *M*(0, *β_opt_*(0)), “NGPA” = *M*(*α_opt_*, *β_opt_*).

Using the definition (Equation 7), we calculated the Gromov hyperbolicity for connectomes and model networks. The results are shown in Figure 3 right (lower values of *δ*(*G*) correspond to “larger hyperbolicity”). We found that structural connectomes exhibit pronounced hyperbolic properties. Model *M*(*α_opt_*, 0) had the lowest hyperbolicity index, which is due to its highly heterogeneous structure and dependence on a small set of superhubs that other nodes connect to. Other models, including NGPA, did not replicate the observed hyperbolic behavior of the connectome, though the NGPA model came closest. We attribute this to the rich-club organization in human connectomes (Figure 3, left), which the NGPA lacks (see the Discussion section).

The rich-club coefficient is defined as

$$\phi \left(k\right)=\frac{2E_{>k}}{N_{>k}\left(N_{>k}-1\right)}$$ (8)

It measures the “network core” density, which is known to be high in brain networks (van den Heuvel & Sporns, 2011) and claimed to be important for efficient information processing.

## STATISTICS OF EIGENMODES

### IPR_2_ Across the Spectrum

Let us turn to a deeper study of the localization properties of eigenmodes. Traditional indicators for localization or delocalization of eigenmodes include: (i) the level spacing distribution *P(s)* and (ii) the IPR. In our previous work (Pospelov et al., 2019), we exclusively focused on *P(s)* and discovered a hybrid Wigner-Poisson distribution in the continuous region, alongside a deformed Poisson distribution in the cluster-related, low-energy segment of the spectrum.

The level spacing distribution, *P(s)*, serves as an integral descriptor of the spectrum. Therefore, to investigate the localization properties of individual modes, it is useful to focus on the IPR. In this discussion, we direct our attention to the IPR of individual eigenmodes of the adjacency matrix *A*; large IPR values signify localized states. Subsequently, we denote by ${IPR}_{q}^{\left(i\right)}$ the IPR for the eigenvector *ψ_i_*(*n*) where *i* counts the eigenvectors and *n* denotes the *n^th^* component of the eigenvector *ψ_i_*(*n*) = {*ψ_i_*(1), *ψ_i_*(2), …, *ψ_i_*(*N*)}:

$${IPR}_{q}^{\left(i\right)}=\sum_{n=1}^{N}{\left|\psi_{i}\left(n\right)\right|}^{2q}$$ (9)

**Figure 4.**
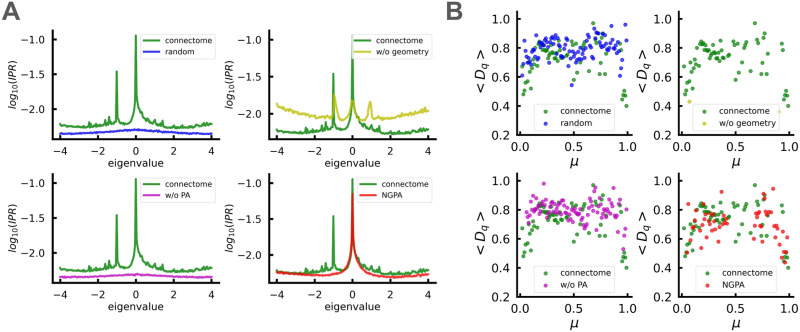
(A) IPR_2_ of the adjacency matrices’ eigenvectors as functions of corresponding eigenvalues. (B) Average fractal dimensions of eigenmodes with a certain position *μ* on the spectrum (*μ* is defined as $\mu_{i}\left(\lambda_{i}\right)=\frac{i}{N},i\in \left[1,N\right]$; see the Methods section for details of multifractal eigenmodes detection). Data are shown for connectomes and corresponding network models and are averaged over 100 connectomes. Models shown: “random” = *M*(0, 0), “w/o geometry” = *M*(*α_opt_*, 0), “w/o PA” = *M*(0, *β_opt_*(0)), “NGPA” = *M*(*α_opt_*, *β_opt_*).

**Figure 5.**
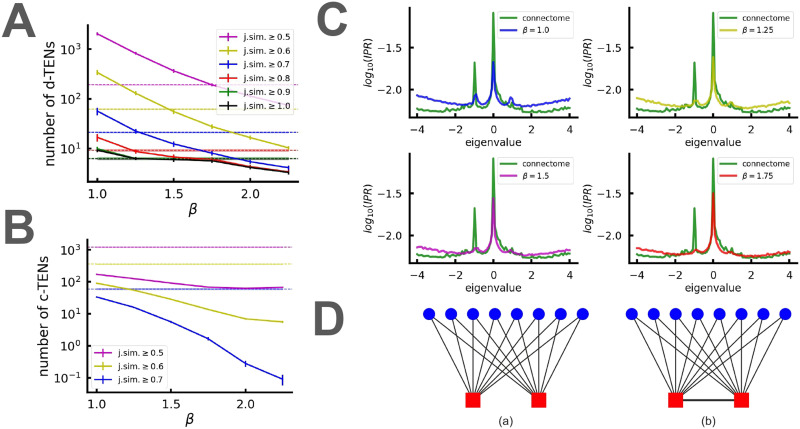
(A) Number of disconnected TENs in NGPA models as a function of geometric parameter *β*. Different curves correspond to different TEN criterions (goodness of a TEN is measured via Jaccard similarity). Dashed curves of the same color correspond to numbers of TENs in the corresponding connectomes with the same TEN criterion. (B) The same for connected TENs. (C) IPR_2_ of the adjacency matrices’ eigenvectors as functions of corresponding eigenvalues for connectomes (green) and NGPA models with fixed *α* = *α_opt_* = 3 and different geometric constants *β*. (D) Examples of TENs (red squares) in the network (blue circles). A disconnected (a) and connected (b) TEN pair are shown. Disconnected TENs correspond to a peak in IPR at *λ* = 0; linked TENs correspond a peak in IPR at *λ* = −1.

Mixed statistics as reported in Pospelov et al. (2019) indicate the presence of localized states within the bulk of the spectrum. Analyzing the IPR_2_, we did identify such states. However, their nature is distinct: They exhibit significant peaks at *λ* = 0 and *λ* = −1 in the spectrum of adjacency matrix as depicted in Figure 4A. Notably, the positions of these peaks are not arbitrary; they correspond to scar-like localized states in the continuous part of the spectrum, which have been extensively studied in Kochergin et al. (2023). The existence of these states has been revealed within networks containing TENs, which possess similar connectivity to their surroundings. It was argued that due to the specific symmetry property of these modes, they are located in a spectrum at *λ*_*n*_ = ± $\sqrt{n},n\in Z$. The TEN nodes corresponding to *λ* = 0 are not connected to each other; hence, some of the *λ* = 0 states are trivial TENs localized at the ends of the leaves that evidently have the similar surrounding. Such states have been previously discussed for heterogeneous networks (Silva & Metz, 2022; Tapias & Sollich, 2023). However, some of *λ* = 0 states are localized at a small amount of nodes in the bulk of a connectome. A connected (interacting) pair of TENs gives rise to a peak at *λ* = −1 (Kochergin et al., 2023) and the complicated TEN complexes, which are quite rare, gives rise to the states with *n* > 1. In cases where TENs are nonideal, a distribution of large IPR values around *λ* = {0, 1} emerges, as is explained in Kochergin et al. (2023). The examples of TENs are presented at Figure 5D.

Figure 4A illustrates perfect identification of the *λ* = 0 peak, while the *λ* = −1 peak is absent in the NGPA model. The possible explanation for this discrepancy is as follows: As we mentioned above, the *λ* = −1 peak corresponds to connected TENs at some distance. If the cutoff scale introduced is smaller than the typical distance between interacting TENs, they are artificially suppressed, leading to the absence of the *λ* = −1 peaks. To test whether this hypothesis is valid, we looked for emergence of *λ* = −1 peaks by increasing the cutoff scale. As expected, these peaks appeared when the cutoff scale was increased by a factor of 3–4 (see Figure 5C), demonstrating that the typical scales responsible for averaged spectral density and correlations between individual TENs differ slightly. To validate these results further, we directly computed the number of disconnected (Figure 5A) and connected (Figure 5B) TENs for various values of *β* in the NGPA model (in all experiments here *α* = *α_opt_* = 3) and corresponding connectomes. To identify whether a given pair of nodes is a TEN pair or not, we calculated Jaccard similarity index for their connectivity sets. That is, given a node *i* with connectivity (set of neighbors) *A* and another node *j* with connectivity *B*, they are considered a TEN pair if $J\left(A,B\right)=\frac{|A\cap B|}{|A\cup B|}>J_{0}$, where 0 ≤ *J*_0_ ≤ 1 is a selected threshold. *J*_0_ = 1 corresponds to ideal TENs (Figure 5D), which are rare. Therefore, we present results for various *J*_0_ thresholds indicating different criteria of TEN “softness.” The results obtained show that the number of disconnected TENs in connectomes can be effectively reproduced by selecting *β* in the range [1.0,1.75] depending on the TEN criterion chosen. At the same time, the number of connected TENs cannot be reproduced by the model even for very low values of *β*, instead, *β* ≈ 1 is the region where these TENs start to form in the model in detectable amounts. This process corresponds to emergence of the IPR_2_ peak around *λ* = −1 in Figure 5C.

In addition to specific localized TENs in the continuum part of the spectrum discussed above, we also observe states with significant IPR_2_ in isolated soft modes of the Laplacian corresponding to the clusters. This type of more trivial localized modes has been investigated in Avetisov et al. (2020) and Kochergin et al. (2023). Let us emphasize that these eigenmodes associated with soft modes exhibit localization not at a single cluster but at a few clusters (see the Methods section for visualization).

### Multifractality of Connectome Eigenmodes

Higher IPR*_q_* provide the possibility to investigate the fractality and multifractality of the spectrum. The multifractality can usually be analyzed via the fractal dimension, *D*_*q*_ (see Evers & Mirlin, 2008), for the review. Consider the size *N* dependence of IPR*_q_*

$${IPR}_{q}∼N^{-\tau_{q}},\;\tau_{q}=D_{q}\left(q-1\right),$$ (10)

where the fractal dimension for a given *q* is defined in the interval 0 < *D*_*q*_ < 1. For localized states *D*_*q*_ = 0, and for completely delocalized states, *D*_*q*_ = 1. If *D*_*q*_ has nontrivial *q*-dependence, the state is multifractal in a strict sense. Otherwise, we shall refer to it as a fractal state. One can consider the fractal dimension of individual states as well as the averaged fractal dimension over the spectrum; here, we focus on the former option. In the case of weak multifractality, the fractal dimensions can usually be presented in the form

$$\tau_{q}=d\left(q-1\right)+\gamma q\left(1-q\right),\;\gamma <<1$$ (11)

We investigated the fractal dimensions of eigenmodes in real structural human connectomes and random models. The advantage of the dataset we used was that the structural connectomes were built at different scales, with each one approximately twice the size of the previous one (60, 125, 250, 500, and 1,000 nodes). We excluded the smallest networks with a size of 60 nodes from our analysis of multifractal properties, leaving us with four networks at four different spatial scales to study the dynamics of eigenstates as the system size changed. Details can be found in the “Multifractality Analysis” section in the Methods section.

**Figure 6.**
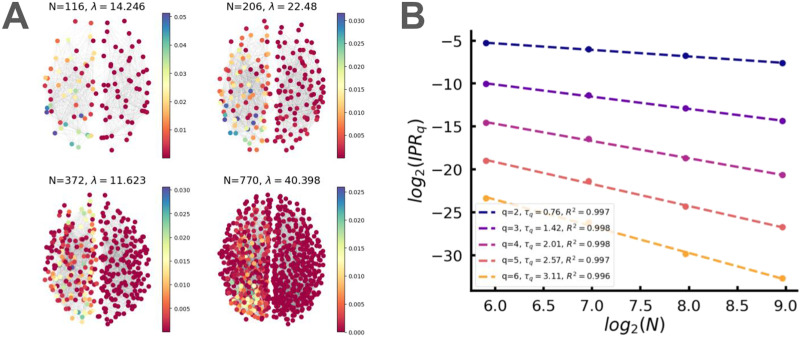
(A) Visualization of a single eigenmode across four different resolution scales. The color encodes the squared eigenvector elements, which represent the probability distribution over nodes. (B) IPR*_q_*(*N*) dependence for different *q* values, along with the corresponding linear fit. The values of *τ_q_* are shown in the legend.

**Figure 7.**
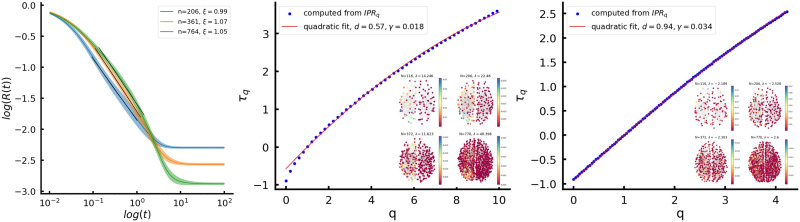
Left: power-law region in the return probability *R(t)* across three different scales. Curves are averaged over 100 networks, for all scales, we find *ξ* ≈ 1. Central: *τ_q_*(*q*) dependence for a single out-of-bulk eigenmode localized in the left hemisphere. Right: the same for an in-bulk eigenmode.

First, we found multiple fractal states in the spectrum with a clear-cut linear dependence for *τ*(*q*) (see Figure 6). These states are mostly distributed in the bulk of the spectrum and have a nontrivial distribution of the fractal dimensions *D*_*q*_(*λ*). Next, we looked for deviations from linearity in *τ*(*q*) to confirm multifractality. Assuming a quadratic fit Equation 11, we have found multiple multifractal states with small *γ* and parameter *d* depending on the position of the eigenmode in the spectrum. Figure 7 shows examples of typical eigenmodes along with their corresponding *τ_q_*(*q*) plots. The quadratic fit confirmed that the system was in a weak multifractal regime (*γ* < < 1) and also showed lower values of *d* for eigenmodes modes outside the bulk corresponding to clusters (see also Figure 4B).

One more indication of a multifractal network state is the power-law in the return probability *R(t)* defined as

$$R\left(t\right)=\frac{1}{N}\sum_{i}e^{-t\lambda_{i}^{lap}}\;R\left(t\right)∼t^{-\xi }$$ (12)

where $\lambda_{i}^{lap}$ is the *i*-th eigenvalue of the network Laplacian *L*. We have found the power-law scaling for *R(t)* with *ξ* ≈ 1 (see Figure 7), which suggests that the decay of eigenstates is slower than it would be in a fully delocalized system.

### Correlation of Cluster Modes

**Figure 8.**
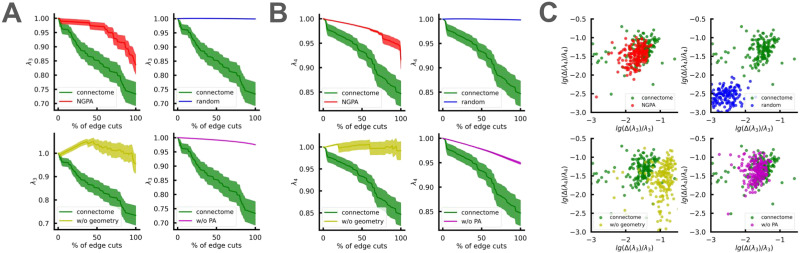
(A) *λ*_3_ dynamics as interhemispheric edges are progressively removed from the connectomes and corresponding random models. The bars indicate standard deviations calculated from 100 connectomes. (B) The same for *λ*_4_ dynamics. (C) Relative changes of *λ*_3_ and *λ*_4_ after removing all interhemispheric edges from connectomes and model networks. Each dot represents a single network. Models shown: “random” = *M*(0, 0), “w/o geometry” = *M*(*α_opt_*, 0), “w/o PA” = *M*(0, *β_opt_*(0)), “NGPA” = *M*(*α_opt_*, *β_opt_*).

Our results on deformed Poisson distribution for the soft modes found in Pospelov et al. (2019) indicate that the community structure of connectomes may lie in the realm of interacting clusters. To validate the presence of correlations among the soft modes of the normalized Laplacian *L*^norm^, we study the behavior of its ordered eigenvalues *λ*_3_ and *λ*_4_ as *λ*_2_ varies. Recall that *λ*_2_ is linearly proportional to the minimum number of links necessary to partition the network into two disjoint components (Chung, 1997). In our context, *λ*_2_ approximately counts the number of connections between the two hemispheres of the brain (M. B. Wang et al., 2017). As expected, the number of clusters correlates with the number of isolated soft modes of the Laplacian. To test their independence, we progressively cut interhemispheric edges in connectomes and random models and investigate the dynamics of *λ*_3_ and *λ*_4_ (see Figure 8).

We observe that the *M*(0, 0) random model fails to accurately reproduce the correlation among soft modes, with changes in *λ*_3_ and *λ*_4_ being ≈10 times weaker than in real networks. At the same time, both geometry-aware models qualitatively reproduce the effect (although NGPA superiority is clear on *λ*_3_ dynamics; see Figure 8). The explanation for the symmetry-breaking behavior of the *M*(*α_opt_*, 0) model, as well as geometric distribution of the first normalized Laplacian eigenvectors, is given in the Methods section (Visualization of Laplacian Eigenvectors section).

Thus, we conclude that anomalous coupling between cluster modes (as compared with a random network) does really exist. We also find that this coupling is geometry-dominated, arising from the limited edge lengths and resulting local hemispherical structure.

## DISCUSSION

It is known that the brain seeks to find an optimal balance between the efficiency of information processing, which requires a large number of connections, and the efficient use of metabolic resources needed to maintain these connections (Bullmore & Sporns, 2012). Various topological features of brain networks, such as the small-world structure and the presence of highly connected hubs, are consequences of the need to maintain this equilibrium. In this study, we discuss a simple two-parameter model of structural connectomes that incorporates both of these aspects and is able to replicate many of the characteristics of real-world brain networks.

The discussed generative model, NGPA, integrates the stochastic nonlinear preferential attachment model with the exponential geometrical model, incorporating an intrinsic spatial scale denoted as *r*_0_. By comparing the results of our model to real data, we determined that *r*_0_ ≈ 3.5 *mm* within the structural human connectome. Our research further supports the significance of metric aspects in brain architecture. To some extent, the NGPA model enhances wiring efficiency and information processing efficiency simultaneously. The model reproduces several key network properties, although it does not properly capture the hyperbolic embedding properties or the *IPR*_2_ peak at *λ* = −1 (which can be reproduced by increasing *r*_0_ by a factor of 3 to 4, suggesting a typical scale for interacting TENs).

One possible explanation for the failure of the NGPA model to produce a good hyperbolic embedding of connectomes may be the existence of a “rich club” of nodes in the brain network (van den Heuvel & Sporns, 2011). These “rich club nodes” are densely interconnected and play a crucial role in providing the shortest paths in the network, creating a heterogeneity among nodes based on their significance in information transfer. At the same time, the formation of a “rich club” is not accounted for in the NGPA model, which may lead to a more even distribution of node participation in signal transmission, despite the structural diversity present in the network. This immediately leads to an increase in Gromov hyperbolicity, since it is a measure of “democracy” in nodes’ participation in optimal paths (Borassi, Chessa, & Caldarelli, 2015).

In this work, we explored the possibility of using preferential attachment to create hubs and construct the densely connected structural core of a network. While this was partially achieved, as shown in Figure 3, this attempt was not fully successful. One reason for this may be due to the dissortative nature of the Barabási-Albert model with *α* > 1, which is inherent in the network (Onody & de Castro, 2004). To improve the NGPA model, a promising approach would be to replace preferential attachment with a more physically plausible principle for creating node heterogeneity. One such approach would be creating a hub by nonlinearly expanding the network (Bauer & Kaiser, 2017).

Our current investigation expands on the analysis of the localization of structural connectome modes initiated in Moretti and Muñoz (2013) and Pospelov et al. (2019). The spectrum includes soft cluster modes and a bulk continuum. States corresponding to the soft modes are localized within clusters. In agreement with the previous arguments in Pospelov et al. (2019), we observed nontrivial correlations between clusters, which explain the semi-Poissonian level spacing behavior in the cluster bands found there.

A notable observation from Pospelov et al. (2019) is the mixed Wigner-Poisson behavior of *P(s)* in the continuum, suggesting that the brain operates near a critical regime with a mobility edge that distinguishes localized and delocalized modes within the bulk of the spectrum. While we qualitatively support the findings of Pospelov et al. (2019), which suggest the existence of localized modes in the bulk, we recognize that the interplay between these localized and delocalized modes is more complex. We investigated the IPR_2_ of individual modes and qualitatively supported the former observation concerning the presence of both localized and delocalized modes in the bulk. However, the distribution of these localized states is more subtle. The majority of them are scar-like states originating from nonideal noninteracting TENs, which form a tiny band around *λ* = 0 in the adjacency matrix spectrum. Some of the localized states correspond to the trivial TENs arising from localization at the ends of the graph leaves, while others are clear-cut localized states at *λ* = −1, corresponding to interacting TENs. The boundary between these localized states and the rest of the delocalized modes in the continuum is not sharp, so there is no strict mobility edge in the bulk as anticipated in Pospelov et al. (2019).

The analysis of IPR*_q_* reveals one more remarkable feature of the structural connectome. It turns out that there are multiple fractal states distributed throughout the spectrum. Furthermore, we have found signs of weak multifractality due to the quadratic dependence of *D*_*q*_(*q*). The power-law behavior of the return probability we found is another indication of multifractal states.

The clear-cut fractal nature of eigenmodes and the hallmarks of multifractality lead us back to the question of the criticality of the structural connectome, specifically the relation to the Anderson localization of eigenmodes. There are two possible scenarios: (i) a critical point at a fixed value of a control parameter or (ii) a phase over a range of control parameters. The latter option is more desirable due to its lack of fine tuning, which seems much more likely from a biological standpoint. Two options host the stretched Anderson critical point: the Griffiths phase and the extended non-ergodic phase (NEE phase). These are considered to be distinct, despite having some similarities.

In both cases, it is expected that heterogeneity is the source of the critical phase; however, the selection of the proper measure of heterogeneity as the control parameter is not evident. In the Griffiths phase, the key factors are the rare effects that correspond to spectral edges, similar to Lifshitz tails or isolated cluster eigenvalues for the graph Laplacian. The power-law behavior is found in some interval of the control parameter. The NEE phase has a typical wavefunction with a few maxima located at several points in the whole system, manifesting multifractality, and the fractal dimension *D*_*q*_ is *q*-dependent. A possible mechanism for the NEE phase’s existence is the presence of minibands in the bulk B (Altshuler & Kravtsov, 2023; Khaymovich, Kravtsov, Altshuler, & Ioffe, 2020). At large *N*, the number of states in a miniband diverges while its width decreases, resembling our spectra with a peak at *λ* = 0 in the spectral density (see Figure 1). The origin of the NEE phase within the framework of the renormalization group was discussed in Zirnbauer (2023) and B. L. Altshuler, Kravtsov, Scardicchio, Sierant, and Vanoni (2024).

Turning back to our study, let us note that we do not have any tool to analyze connectomes analytically, but the spectral properties we found numerically are suggestive. Indeed, we have a number of separated localized cluster-related modes as well as peak at *λ* = 0 in the bulk, populated by localized modes with a high IPR_2_. Additionally, there is a clear-cut IPR peak at *λ* = −1. The cluster modes interact with each other, yielding non-Poisson statistics in the cluster band (see Figure 8). Therefore, these findings are similar to those reported in Cugliandolo et al. (2024), and indeed, the fractality of multiple modes is identified. At least half of the extended modes are fractal with a typical fractal dimension *D*_2_ = 0.7 − 0.8 (see Figure 4B, note that both modes outside the bulk and those around *λ* = 0 have significantly lower *D*_*q*_). More refined fitting for *D*_*q*_ reveals weak multifractality, although the deviation from the linearity is small. We also find power-law behavior in the return probability, analogously to Cugliandolo et al. (2024). Of course, the architectures of the connectome and the model in Cugliandolo et al. (2024) differ since we are not dealing with a sparse case.

Therefore, to make any firm statement concerning the relevance of the NEE phase for the structural connectome, we need to introduce a proper control parameter that measures heterogeneity, like the one considered in Silva and Metz (2022), and identify a possible critical interval for that control parameter. This point definitely requires detailed study. There are many reservations about the idea that the connectome operates in a nonergodic extended phase. For one thing, the size of the system is small, and we cannot rule out the possibility that weak multifractality in the spectrum is a finite-size effect. Also, the analysis only involves four points at different *N*, and the directions of connectome links are not considered. Nevertheless, despite all these subtleties, we suggest that the structural connectome does operate in the NEE phase, although it might be very close to the Griffiths phase within its general framework.

Concerning the practical aspect of fractality, we can expect that fractal and scar-like structures play specific roles in the propagation of excitations within the brain, with scar-like modes residing predominantly in the bulk part of the Laplacian spectrum. Specifically, if the initial state significantly overlaps with a scar-like state, long-living oscillations at the corresponding frequency may emerge. The fractality of the modes in the bulk is believed to slow down decay processes due to a power-law relation for the return probability, closely related to the decay rate.

Exploring the information theory aspects of our model, including various entropic measures, as discussed in Bianconi (2021), would be intriguing. For example, recently, the relation between fractal properties of the spectrum and the entanglement entropy of two subsystems has been explored in De Tomasi and Khaymovich (2020). It has been argued that under some additional assumptions, the entanglement entropy saturates at *D* > 1/2 generalizing the well-known Page saturation of the subsystem entropy. In our case, we have *D* > 1/2 indeed and natural separation of the system into two hemispheres. It would be interesting to investigate this in more detail.

For the sake of computational tractability and better eigenmode interpretability, we have discarded edge weights in the connectomes under study. This simplification was quite reasonable for the dataset used here, since only consensus-based edges were left in the graphs computed from MRI data (Kerepesi et al., 2017). However, axonal tracts may vary significantly in thickness, allowing for different information transmission bandwidths. For example, weight distributions in large-scale mammalian connectomes have been shown to span multiple orders of magnitude (Theodoni et al., 2021), in full agreement with the “log-dynamic brain” theory (Buzsáki & Mizuseki, 2014). We intend to account for the influence of edge weights on network dynamics and compare the results to other generative models producing weighted connectomes (Akarca et al., 2023; Goulas et al., 2019) in future publications.

Special attention should be paid to edge directions. Despite the fact that there are many bidirectional connections in the brain that play an important functional role, such as providing stable zero-lag synchronization between cortical nodes (Gollo, Mirasso, Sporns, & Breakspear, 2014), for a complete understanding, it is also important to consider the direction of information transmission along axonal tracts. For example, it has been shown that in-weights gradients in the cortex determine traveling wave direction (Koller, Schirner, & Ritter, 2024). The analysis of directed brain networks involves dealing with asymmetric adjacency matrices and complex spectra, which makes mathematical analysis difficult and limits the possibilities for theoretical understanding. Nevertheless, even in the simplified random neuronal non-Hermitian model (Wardak & Gong, 2022), interesting behavior has been found for the heterogeneous heavy-tailed connectivity. The marks of the extended Anderson criticality with the diversity of the timescales have been discovered. We intend to extend our analysis to directed connectomes in forthcoming publications.

## METHODS

### Human Connectome Data

We used the braingraph.org database (Kerepesi et al., 2017) for our analysis. This database contains structural connectomes of 426 human subjects computed from high-angular resolution diffusion imaging data from the Human Connectome Project (McNab et al., 2013). Each connectome has been constructed at five different resolutions, and we used the graph of the highest resolution (with 1015 nodes) for further analysis, except for the investigation of multifractal properties, which required multiple resolution data. Each connectome was preprocessed in the following way: (i) Isolated nodes and self-loops were removed; (ii) for graphs with more than one connected component, only the largest one was kept. Connectomes in which the largest component covered less than 75% of the nodes were excluded from the dataset; (iii) edge weights were discarded, and binary adjacency matrices were constructed.

We extracted the coordinates of all nodes for further construction of spatially embedded model networks from each connectome in the dataset. We ensured that the Euclidean distances between nodes connected by an edge correlate well with the actual length of the axonal fibers in the dataset (Pearson’s correlation coefficient *r* = 0.91). Therefore, we verified the proxy relationship between the real and Euclidean distances (Kaiser, 2011) and used the latter for our further analysis. This enabled construction of random geometry-aware connectome models.

The random models were constructed for each connectome separately.

### Selecting Optimal Parameters for NGPA Model

**Figure 9.**
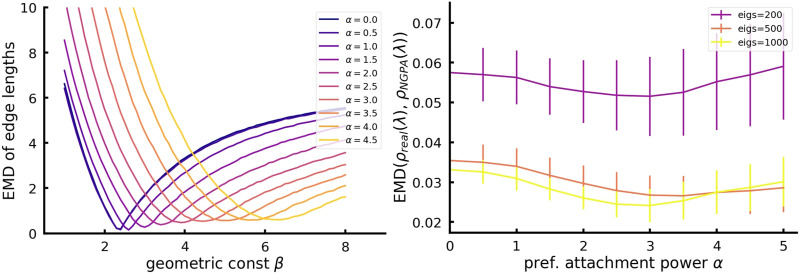
Left: EMDs between edge length distributions as a function of *β*. Each curve corresponds to a different *α* value. Right: EMDs between the normalized Laplacian spectrum of connectomes and their corresponding NGPA models, as a function of *α*. The three curves are for distances calculated from the first 200, 500, and 1,000 eigenvalues, respectively. The curves are averaged over 100 networks (connectomes and their corresponding NGPA models).

To select the optimal parameters for the model, we followed a two-stage process. In the first stage, we determined the optimal geometric coefficient, *β*_opt_(*α*), for each potential value of *α*, where our search space was *α* ∈ [0, 5] with step 0.5 and *β* ∈ [0, 8] with step 0.1; see Figure 9 for details. In the second stage, we selected the optimal value *α*_opt_ that minimized the “earth mover’s distance” (EMD; Rubner, Tomasi, & Guibas, 1998) between spectral densities of normalized Laplacian matrices from real connectomes and their NGPA equivalents, with corresponding optimal geometric constraints, *M*(*α*, *β*_opt_(*α*)).

The reasons to use EMD instead of Kullback–Leibler divergence, which is a default choice for comparing probability distributions, are:

1. EMD is a true metric (i.e., it is a nonnegative symmetric function that satisfies the triangle inequality and the axiom of coincidence (Royden, 1988), while KL divergence is not.
2. EMD respects geometry of the metric space, so that it penalizes the shifts of probability density function (Rubner et al., 1998). This is important for network measures, since we not only want to take into account the difference of two probability distributions in terms of relative entropy, but the regions of the support where the differences occur.
3. EMD works well on discontinuous supports, which can occur during empirical data analysis, while KL divergence is undefined on them.

We refer to the comprehensive discussion on probability distribution measures in Gibbs and Su (2002).

The described two-step procedure allowed us to reduce the computational complexity in order to avoid overfitting, while ensuring that the geometric structure of the optimal network coincides with that of the real connectome. In both cases, we used the EMD as a measure of distance between distributions.

Both quantities exhibit a clear minimum, with a near-perfect match between edge length distributions at *β_opt_* (see Table 1 for quantitative results). For further analysis, we use an optimal NGPA model with *α_opt_* = 3 and *β_opt_* = 4.5, which leads to a characteristic geometric scale of approximately 3.5 mm (see Equation 2). The optimal *β* value for a geometry-based model (with *α* = 0) was equal to *β_opt_*(0) = 2.4.

**Table 1.** EMD values indicating distances between corresponding distributions in connectomes and models

EMD distances between distributions				
Network characteristic	Random	W/o geometry	W/o PA	NGPA
Spectral density	0.0450 ± 0.0003	0.071 ± 0.001	0.0336 ± 0.0003	**0.0248** ± **0.0004**
Topological similarity	0.0668 ± 0.0008	0.2404 ± 0.0012	0.0497 ± 0.0007	**0.0152** ± **0.0003**
Clustering coefficients	0.542 ± 0.001	0.204 ± 0.002	0.430 ± 0.001	**0.076** ± **0.001**
Edge lengths	15.57 ± 0.13	20.11 ± 0.14	**0.37** ± **0.01**	0.40 ± 0.02

### Visualization of Laplacian Eigenvectors

**Figure 10.**
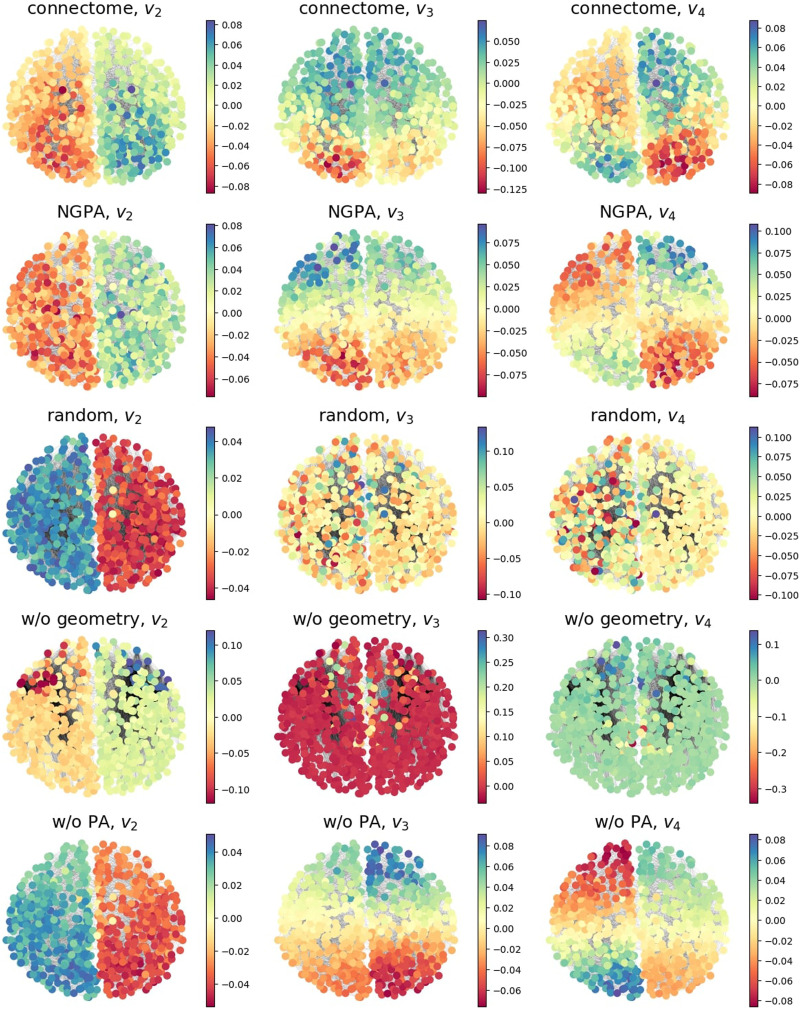
Localization of *L*^norm^ first eigenvectors of one real connectome and corresponding random models. Color encodes vector elements. Models shown: “random” = *M*(0, 0), “w/o geometry” = *M*(*α_opt_*, 0), “w/o PA” = *M*(0, *β_opt_*(0)), “NGPA” = *M*(*α_opt_*, *β_opt_*).

To better show the role of the first normalized Laplacian eigenvectors in connectomes and artificial models, we visualized the first three of them (*v*_2_, *v*_3_, and *v*_4_) in Figure 10. The constant eigenvector *v*_1_ corresponding to $\lambda_{1}^{\mathrm{norm}}=0$ is omitted.

In all models except for *M*(*α_opt_*, 0), the first nontrivial eigenvector *v*_2_ divides the graph into two hemispheres, in full accordance with the idea of minimal cut in spectral clustering (von Luxburg, 2007). The following eigenvectors *v*_3_ and *v*_4_ are localized in single hemispheres and divide them into approximately equal parts. For real connectomes and NGPA models, these parts are clearly localized, since due to the limited edge lengths, the optimal cut lies approximately at the middle of each hemisphere. This feature is especially pronounced in the model without preferential attachment, where the heterogeneity of node degrees does not violate the geometric separation of the hemispheres. Note, however, that in real connectomes and NGPA model, the eigenvector participation in hemisphere separation is mixed. This is another manifestation of stronger interaction between these eigenmodes, which explains better NGPA performance in reproducing *λ*_3_ and *λ*_4_ dynamics during interhemispheric edge cuts (see Figure 8).

The effect of node heterogeneity is revealed in the behavior of eigenvectors in model *M*(*α_opt_*, 0). The presence of hubs in the network distorts the geometric distribution of eigenvector elements, forcing them to concentrate on highly connected nodes. This breaks the usual symmetry between *v*_3_ and *v*_4_, and is the reason for the asymmetry in relative Δ*λ*_3_ and Δ*λ*_4_ for this model in Figure 8.

### Multifractality Analysis

For convenience, in this section, we use the relative spectrum

$$\mu_{i}\left(\lambda_{i}\right)=\frac{i}{N},\;i\in \left[1,N\right]$$ (13)

When analyzing structural connectomes for the multifractality of eigenstates, we encountered several technical challenges. First, in contrast to artificial systems, working with brain networks means that we do not have the ability to generate new data quickly. Instead, we must work with a limited set of previously collected networks (Kerepesi et al., 2017). Second, even the highest resolution networks are significantly smaller than the systems in which multifractal behavior is typically studied (see, e.g., Roy, Khaymovich, Das, & Moessner, 2018). Finally, the high level of individual variability in brain networks makes it difficult to apply simple heuristics for finding corresponding eigenmodes in systems of different sizes. For example, it is not possible to simply take an eigenvector that corresponds to the same normalized eigenvalue *μ_i_*.

To find the corresponding eigenstates in networks of different sizes, we used the following procedure:

▪  Each eigenvector *v*_*j*_ was projected onto an 87-dimensional “anatomical space” in which each anatomical area of the brain (region of interest [ROI]) was represented by a separate dimension. The elements of this vector were calculated as $a_{i}=\sum_{k}{\left(v_{j}^{2}\right)}_{k},k\in {ROI}_{i}$. In other words, *a*_*i*_ contained aggregated ROI-related probabilities induced by the eigenvector *v*_*j*_, summed over all anatomical subdivisions of this ROI on a given connectome resolution.
▪  The Jensen-Shannon divergence (JSD) was calculated between these “anatomical” vectors, as a measure of the information dissimilarity between two probability distributions over the ROIs. In the subsequent analysis, only groups of vectors with JSD values of ≤0.4 were considered (since multifractal properties were analyzed using data at 4 scales, a total of $C_{4}^{2}=6$ JSD values were calculated).
▪  Additionally, we imposed a restriction on the relative positions of the eigenmodes in the spectrum: The relative eigenvalues at adjacent scales *S* and *S* + 1 should be close to each other: $|\mu_{i}^{S}-\mu_{i}^{S+1}|<0.05$.

For the groups of eigenmodes selected in this way, the function IPR*_q_*(*N*) was calculated for different values of *q*. Only eigenmodes with a good linear fit to this function on a log–log plot (*R*^2^ > 0.95 for every *q*) were considered for multifractality analysis.

## ACKNOWLEDGMENTS

We are grateful to I. Cheryomushkin for collaboration at the early stages of the project, as well as to A. Brazhe and K. Anokhin for useful discussions. N.P. acknowledges support from the Non-Commercial Foundation for Support of Science and Education “INTELLECT.” A.G. thanks IHES, where the part of the work has been done, for the hospitality and support.

## AUTHOR CONTRIBUTIONS

Anna Bobyleva: Conceptualization; Data curation; Investigation; Software; Visualization; Writing – original draft. Alexander Gorsky: Conceptualization; Formal analysis; Funding acquisition; Project administration; Supervision; Validation; Writing – original draft; Writing – review & editing. Sergei Nechaev: Formal analysis; Funding acquisition; Project administration; Supervision; Writing – original draft. Olga Valba: Data curation; Methodology; Software; Writing – original draft. Nikita Pospelov: Conceptualization; Data curation; Investigation; Methodology; Resources; Software; Validation; Visualization; Writing – original draft; Writing – review & editing.

## FUNDING INFORMATION

Nikita Andreevich Pospelov, Non-Commercial Foundation for Support of Science and Education “INTELLECT” (https://dx.doi.org/10.13039/100007857).

## DATA AND CODE AVAILABILITY

Analysis of connectomes and construction of model networks were performed in Python. The analysis code relies heavily on the DRIADA package (Pospelov, 2024), aiming at network and neuronal population-level analysis of brain activity. Some parts of this research were completed using the NetworkX package (Hagberg, Swart, & Schult, 2008). The data used can be found at braingraph.org database (Kerepesi et al., 2017). Full code for network analysis and the scripts producing all figures can be accessed via Google Colab.

## TECHNICAL TERMS

Level spacing distribution: Is a concept in quantum mechanics and statistical physics, particularly in the study of random matrix theory. It refers to the probability distribution of the spacings between adjacent eigenvalues (energy levels). The study of level spacing distributions helps in understanding the nature of quantum systems, including their transition between chaotic and regular behavior.
Inverse participation ratio (IPR): Is a measure used to quantify how localized or delocalized a state is in a quantum or statistical system (e.g., on a graph). IPR reflects how a wavefunction is spread across a given basis (e.g., number of nodes in the graph). At low IPR, the wavefunction is delocalized, spread over many nodes. At high IPR, the wavefunction is localized, concentrated in a few nodes. It is commonly used in the study of localization phenomena, such as Anderson localization.
Multifractality: Refers to a complex type of scaling behavior found in systems where the system has different fractal dimensions across different scales. In physics, multifractality often appears in disordered systems at critical points in phase transitions. It is characterized by a spectrum of exponents, unlike simple fractals that can be described by a single scaling dimension. Multifractality provides a detailed description of systems with varying degrees of local irregularity.
Anderson localization: Is a phenomenon in condensed matter physics and in physics of disordered systems where a particle (or excitation) gets trapped and unable to move through a disordered medium. This leads to the localization of the particle’s wavefunction in space. It occurs when the disorder in the system, such as impurities in a material or randomness in a network, is strong enough to prevent the particle from propagating through the system, causing it to stay localized in some region.
Mobility edge: Is an energy threshold in disordered systems that separates localized states from delocalized states in the context of Anderson localization. Below the mobility edge, states are localized and excitations are trapped in specific regions of the system. Above the mobility edge, states are delocalized, allowing particles to move freely through the system. The mobility edge marks the critical energy at which a system transitions from localized to delocalized behavior.
Griffiths phase: Is a phase observed in disordered systems in a narrow region around the critical point of the phase transition. In the Griffiths phase, some parts of the system behave as if they are in the ordered phase, even though the overall system is disordered. This leads to abnormally slow dynamics, nonuniversal critical behavior, and “stretched exponential,” or power-law behaviors in correlations.
The non-ergodic extended (NEE) phase: Refers to a phase in disordered systems where wavefunctions are delocalized but do not fully explore the available phase space, violating the ergodicity principle. Key characteristics of the NEE phase are: (a) delocalization; (b) non-ergodicity (the system does not equilibrate or explore all possible phase space); (c) intermediate between phases (NEE phase lies between a fully Anderson-localized and a fully ergodic extended phases).
Rosenzweig-Porter (RP) model: Is a simple random matrix model used to study transitions between different phases of localization and ergodicity in the context of disordered systems. It generalizes the classical random matrix theory by introducing tunable “diagonal” disorder that can create different phases. RP model has three phases: (a) localized phase; (b) non-ergodic extended (NEE) phase; (c) ergodic extended phase.
Topologically equivalent nodes (TENs): Refer to nodes in a network or graph that share the same topological characteristics or structural properties. TENs have the following properties: (a) They are indistinguishable in terms of their connectivity to other nodes in the network; (b) they play equivalent roles in the network’s structure and function; (c) any operation on one of the TENs can be applied to the others without changing the graph’s topology. TENs are used in graph theory and network science to simplify complex structures by identifying symmetries of subsystems. Two different ideal TENs are schematically depicted in Figure 5D, where blue circles designate network vertices, and red squares represent vertices that are TENs. If two ideal TENs are disconnected as in Figure 5D (a), the IPR has a strong peak at *λ* = 0, while if they are connected as shown in Figure 5D (b), then IPR is peaked at *λ* = −1.
Gromov hyperbolicity (GH): Is a geometric property of metric spaces that generalizes the notion of hyperbolic geometry. It provides a way to characterize spaces that exhibit certain “tree-like structures,” often found in graphs and groups. GH is widely used in various fields, including: (a) in geometric group theory—to study the properties of groups based on their Cayley graphs; (b) in topology—to understand the structure of spaces and their fundamental groups; (c) in graph theory—to analyze properties of graphs that may exhibit hyperbolic characteristics.
Poisson statistics: Emerges in systems that are usually integrable, where energy levels are uncorrelated, resulting in an exponential distribution of spacings between adjacent eigenvalues (no level repulsion).
Entanglement entropy: Is a measure of the amount of quantum entanglement between subsystems in a quantum state. For a bipartite system with a density matrix *ρ_AB_*, the entanglement entropy *S*_*A*_ of subsystem *A* is given by the von Neumann entropy of its density matrix *ρ_A_: S_A_* = −Tr(*ρ_A_* log*ρ_A_*). A higher entanglement entropy indicates greater entanglement between the subsystems.
Wigner-Dyson statistics: Emerges in systems that exhibit chaotic behavior, where energy levels (eigenvalues) tend to repel from each other, leading to a distribution where small spacings are less likely (level repulsion).
Jaccard similarity matrix: Jaccard similarity quantifies the similarity between the sets A and B, it is defined as: $J\left(A,B\right)=\frac{|A\cap B|}{|A\cup B|}$
